# The Dynamic Interplay Between Cardiac Inflammation and Fibrosis

**DOI:** 10.3389/fphys.2020.529075

**Published:** 2020-09-15

**Authors:** Toby P. Thomas, Laurel A. Grisanti

**Affiliations:** Department of Biomedical Sciences, College of Veterinary Medicine, University of Missouri, Columbia, MO, United States

**Keywords:** cardiac, fibrosis, fibroblast, inflammation, leukocyte, cytokine

## Abstract

Heart failure is a leading cause of death worldwide. While there are multiple etiologies contributing to the development of heart failure, all cause result in impairments in cardiac function that is characterized by changes in cardiac remodeling and compliance. Fibrosis is associated with nearly all forms of heart failure and is an important contributor to disease pathogenesis. Inflammation also plays a critical role in the heart and there is a large degree of interconnectedness between the inflammatory and fibrotic response. This review discusses the cellular and molecular mechanisms contributing to inflammation and fibrosis and the interplay between the two.

## Introduction

Cardiovascular disease is the leading cause of death worldwide and represents an immense health and economic burden (Benjamin et al., 2019). It is comprised of a group of conditions affecting the blood vessels and heart, culminating in impaired cardiovascular performance. Heart failure, the clinical manifestation of cardiovascular disease, is characterized by fibrosis, chamber remodeling and a reduction in ventricular compliance. Cardiomyocytes have limited capacity for regeneration thus, injury to the heart, leading to death of cardiomyocytes, results in clearing of dead cardiomyocytes and repair through fibrotic scar tissue replacement. This helps maintain the structural and functional integrity of the heart, but results in impairments in contractility and cardiac function when excessive. Ischemic heart disease is the leading type of cardiovascular disease and results in a fibrotic scar, however, fibrosis is a major contributor to many forms of heart disease and is recognized as a pathological hallmark in the heart (Travers et al., 2016).

Inflammation is a major regulator of the reparative response after cardiac injury. Following injury, there is an acute, intense inflammatory response that is important for initiating healing (Prabhu and Frangogiannis, 2016). Later immune responses promote repair. Proper timing and magnitude of inflammatory responses is critical for normal healing. Persistent inflammation can promote further tissue destruction while insufficient responses prolong the injurious stimuli. Inflammation regulates all aspects of cardiovascular health including cardiac fibrosis. There is a high degree of interconnectedness between immune cells and fibroblasts with each regulating the other’s function. While recently these responses have been increasingly studied, inflammatory events that occur in the heart continue to not be fully understood. This further need to understand the mechanisms of cardiac repair is exemplified by the fact that no large-scale immunomodulatory or anti-inflammatory therapeutic strategies have been successfully translated into clinical practice.

## Cardiac Fibrosis

Cardiac fibrosis is the process of pathological extracellular matrix (ECM) remodeling resulting in abnormal matrix composition leading to impairments in cardiac contractility and function. Fibrosis is involved in nearly all types of heart disease including various ischemic and non-ischemic etiologies (Liu et al., 2017). Initially, ECM deposition is protective and important for wound healing, but excessive or prolonged deposition can lead to impairments in tissue function. Fibrosis leads to a stiffer and less compliant heart, ultimately contributing to the progression of heart failure.

In the mammalian adult heart, cardiomyocytes are organized in a network of parenchymal cells, which includes a large number of fibroblasts, and ECM proteins. The ECM is composed predominantly of fibrillary collagens with type I collagen being the predominant form and type III collagen representing a smaller fraction along with other proteins such as fibronectin and elastin (Rienks et al., 2014). The ECM serves as a scaffold for cells and is also important in transmission of contractile forces in the normal myocardium.

Cardiac fibroblasts are the predominant cell type involved in cardiac fibrosis. They reside in the interstitium, epicardial and perivascular regions of the heart. Studies assessing fibroblast numbers have varied depending on species, technique and markers used, but regardless, it is appreciated that there is an abundant fibroblast population in the heart (Nag, 1980; Banerjee et al., 2007). Due to a lack of fibroblast specific markers, studies involving fibroblast have been difficult and likely represent a heterogeneous population of cells and numbers likely vary depending on the species studied, age and gender (Nag, 1980; Camelliti et al., 2005; Banerjee et al., 2007).

While fibroblasts are plentiful in the non-pathological heart, their function remains poorly understood. Resident fibroblasts originate from the embryonic epicardium (Gittenberger-de Groot et al., 1998). Under normal conditions, fibroblasts contribute to the homeostasis of the heart through the contribution of ECM, which serves as a structural scaffold for cardiomyocytes, distributes mechanical forces and mediates electrical conduction (Travers et al., 2016). Fibroblasts also contribute to matrix remodeling through the production of ECM regulatory proteins including the matrix metalloproteinases (MMPs) and TIMPs. Fibroblasts also have the ability to rapidly respond to alterations in their microenvironment. They are networked into the interstitial and perivascular matrix putting them in a strategic location for serving as sentinel cells to sense injury and trigger reparative responses (Kawaguchi et al., 2011; Diaz-Araya et al., 2015).

In the healthy heart, resident fibroblasts remain in the quiescent state, however, during pathological conditions, these resident fibroblasts and other precursor cells become activated and transdifferentiate into myofibroblasts. The origin of activated cardiac myofibroblasts is less clear with potential sources include resident fibroblasts, vascular endothelium, epicardium, perivascular cells and hematopoietic bone marrow-derived progenitor cells. There is substantial evidence that resident fibroblasts proliferate and activate in response to pathological stimuli (Fredj et al., 2005; Teekakirikul et al., 2010; Ali et al., 2014; Moore-Morris et al., 2014) however, these studies do not discount the possibility of other sources of activated fibroblasts. With the advent of transgenic mouse models, lineage tracing studies are beginning to be used to address this question. Endothelial, epicardial and perivascular cells have been proposed to undergo an endothelial-mesenchymal transition to acquire a fibroblast, pro-fibrotic phenotype. Lineage tracing studies have been performed to identify the contribution of these cell populations to the fibroblast population after injury however, many of the markers used such as Tie2 and vascular endothelial cadherin are not specific to the cell population being studied and immune cells express many of these same markers (Kisanuki et al., 2001; Monvoisin et al., 2006; Zeisberg et al., 2007; Russell et al., 2011; Ali et al., 2014; Kramann et al., 2015). Similarly, hematopoietic bone marrow-derived progenitor cells have also been proposed as a potential source of fibroblasts during pathology. This is due to initial studies using GFP-labeled bone marrow transplants where a large number of GFP-positive cells were located in fibrotic regions after pressure overload and myocardial ischemia (Haudek et al., 2006; Zeisberg et al., 2007; van Amerongen et al., 2008). However, these findings may be due to the presence of inflammatory cells in the fibrotic region and not a transition of these cell populations into myofibroblasts (Yano et al., 2005; van Amerongen et al., 2007). Regardless, CD45-positive cells including monocytes can express myofibroblast markers (Haudek et al., 2006) and it is known that inhibition of monocyte recruitment diminishes the cardiac fibroblast population (van Amerongen et al., 2007). Whether this is due to the importance of early immune responses in the recruitment and activation of fibroblast populations is unknown. However, lineage tracing experiments using the Vav-Cre and other lines suggest minimal contribution of hematopoietic cells to the cardiac fibroblast population (Ali et al., 2014; Moore-Morris et al., 2014, 2018).

The identification of fibrocytes in the circulation has renewed the interest of cells of hematopoietic origin as potential fibroblast contributors (Bucala et al., 1994). Fibrocytes are a unique fibroblast progenitor population that expresses fibroblast markers such as pro-collagen I and vimentin as well as hematopoietic markers (Abe et al., 2001). They originate from hematopoietic stem cells and have been shown to contribute to cardiac fibrosis in several injury models (Mollmann et al., 2006; Zeisberg et al., 2007; Xu et al., 2011).

Regardless of their origin, myofibroblasts appear shortly after injury and have a fibroblast-smooth muscle cell phenotype, with the acquisition of α-smooth muscle actin, contractile functions and enhanced secretion of collagens and other ECM components to promote scar formation. In accordance with their hypothesized sentinel cell function, following insult or injury, there is an upregulation of pro-inflammatory and pro-fibrotic factors in cardiac fibroblasts, which culminates in increases in fibroblast proliferation and the transition to a myofibroblast phenotype.

Myofibroblasts are the major cell type responsible for ECM and secretion. They are characterized by the development of stress fibers and expression of contractile proteins such as α-smooth muscle actin (Frangogiannis et al., 2000b; Santiago et al., 2010). Myofibroblasts secrete collagen and other ECM proteins to preserve the structural integrity of the heart. Failure of the heart to adapt and meet the pressure-generating capacity results in myocardial dysfunction and rupture. After ECM deposition, the tensile strength increases at the site of injury leading to mature scar formation. While these processes are initially adaptive, they can lead to the development of adverse changes in compliance and structure, worsening the progression of heart failure over time. Pathological remodeling is characterized by fibroblast accumulation and excessive ECM deposition. This leads to alterations in the heart’s architecture and has additional consequences on cardiac function. Fibrosis damages cardiac function due to the increased stiffness in the ventricle, producing contractile impairments. ECM and fibroblasts can disrupt the mechano-electric coupling of cardiomyocytes, diminishing cardiac contraction and increasing the risk of arrhythmia. Paracrine signaling from fibroblasts can induce hypertrophy and further cardiac dysfunction. Additionally, apoptosis resistant myofibroblasts can reside in mature scars perpetuating these responses.

**FIGURE 1:**
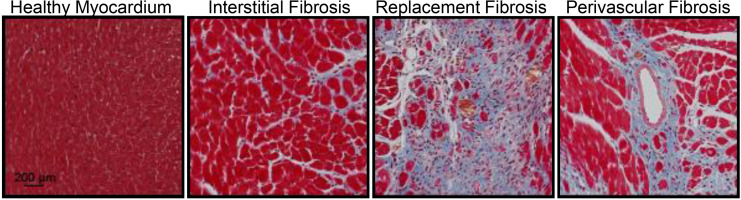
Masson’s trichrome staining of the mouse heart demonstrating fibrosis (blue) in the healthy myocardium, interstitial fibrosis following isoproterenol administration (30 mg/kg/d for 2 weeks), replacement fibrosis after myocardial infarction (4 weeks) or perivascular fibrosis following isoproterenol infusion (30 mg/kg/d for 2 weeks). Red represents the cytoplasm and black represent nuclei. Animal procedures were performed in house with approval by the Institutional Animal Care and Use Committee at the University of Missouri and in accordance to the National Institutes of Health *Guidelines on the Use of Laboratory Animals*.

Cardiac fibrosis presents itself in three forms: perivascular, reactive interstitial, and replacement fibrosis (Anderson et al., 1979), which are exemplified in Figure 1. Reactive interstitial fibrosis is adaptive to preserve cardiac structure and function whereas replacement fibrosis fills areas cause by cardiomyocyte death. Perivascular fibrosis, often occurring with other forms of fibrosis, is characterized by increased collagen deposition around vessels and microvasculature which function to provide oxygen and nutrients to cardiac tissue (Ytrehus et al., 2018). Perivascular fibrosis is heavily involved in hypertension and leads to impaired blood flow hampering the delivery of oxygen and nutrients to potentiate a pathogenic response (Kai et al., 2006). Pressure overload models, such as transaortic constriction, have a period of reactive interstitial fibrosis while the heart adapts to the hemodynamic changes followed by replacement fibrosis upon cardiomyocyte death. During myocardial infarction or ischemia/reperfusion injury, where there is an acute, extensive cardiomyocyte death, replacement fibrosis occurs, which fills the region devoid of cardiomyocytes and prevent cardiac rupture.

Fibrosis is also an essential aspect of cardiac repair. Initially it is a protective process and acts to preserve the architecture of the heart through the deposition of connective tissue. However, fibrosis can become pathological when progressive and excessive, leading to aberrant scarring, further organ damage and impairments in cardiac function. Ischemic diseases, hypertension, valvular disorders, and primary/secondary cardiomyopathies all include at least one of the three types of fibrosis and ECM remodeling (Burt et al., 2014). As the heart adopts a pathologic state, ECM remodeling and excessive fibrosis in turn lead to changes in chamber dimension and in some cases cardiomyocyte hypertrophy. As the injured healing heart adjusts to meet the demands of the rest of the body the myocardium around the fibrotic scar dilates. The progressive increased load on the heart causes a further dilation of the left ventricle, thereby increasing ventricular cavity size. Due to the naturally quiescent non-proliferative state of cardiomyocytes, existing viable cardiomyocytes undergo hypertrophy to account for an increased volume load. These changes lead to progressive ECM remodeling and interstitial fibrosis resulting in decompensated heart failure.

## Inflammation in the Heart

Inflammation is an important defense mechanism that acts to remove harmful stimuli and promote recovery. While some wound healing and fibrotic processes can occur in the absence of cellular immunity, inflammation is an important contributor to cardiac health both in the normal and diseased state. Inflammation of the appropriate timing, duration and magnitude is critical for normal healing. Failure to activate sufficient inflammatory responses can lead to persistence of the injurious stimuli whereas failure to resolve inflammation can further tissue destruction.

Under non-pathological conditions, cardiac macrophages and other resident immune populations help regulate homeostasis. While the role of immune responses in cardiac homeostasis has been an understudied area, it is known that the heart has resident populations of mast cells and macrophages that play an important role in homeostasis and following injury (Sperr et al., 1994; Frangogiannis et al., 1999). There are also small populations of B and T cells present in the healthy myocardium (Pinto et al., 2016). Under steady-state conditions, resident immune cells are believed to play a sentinel role in surveilling against invading pathogens, similar to what is observed in other tissues (Franken et al., 2016). Mast cells are located in the perivascular areas and contain stores of inflammatory mediators such as tumor necrosis factor (TNF), histamine and tryptase, which can be quickly released following injury and represent an important contributor to triggering inflammatory responses (Frangogiannis et al., 1998a; Somasundaram et al., 2005).

The heart also contains resident macrophage populations that are comprised primarily of CCR2- cells of embryonically derived cells that originate from yolk sac macrophages and fetal monocytes (Pinto et al., 2012; Epelman et al., 2014; Heidt et al., 2014; Mylonas et al., 2015). There is also a small population derived from CCR2+ monocytes. Some studies suggest that resident cardiac macrophages die following injury and are replaced by monocyte-derived CCR2-expressing populations that are highly pro-inflammatory (Heidt et al., 2014). Outside of pathogen surveillance, resident immune populations are hypothesized to facilitate physiological turnover of cells and ECM, debris clearance after changes in metabolic load, and also play a role in the conduction system (Swirski et al., 2009). Gene expression profiling has identified distinct profiles of CCR2- macrophages in human myocardium compared to CCR2+ populations with enhanced expression of pathways involved in cell growth and ECM formation (Bajpai et al., 2018). While functional outcomes of these differences of these gene expression differences is not well characterized, these findings are consistent with the role of resident macrophage populations in other tissues (Franken et al., 2016). Outside of the classic role of tissue macrophages, cardiac macrophages have also been recognized as having organ-specific functions. Resident cardiac macrophages are enriched in the conduction system of the heart and depletion disrupts electrical conduction in the heart (Hulsmans et al., 2017). These studies have identified a relationship between cardiomyocytes and macrophages through the formation of gap junctions that enable cardiac macrophages to contribute to steady-state electrical conduction.

**FIGURE 2:**
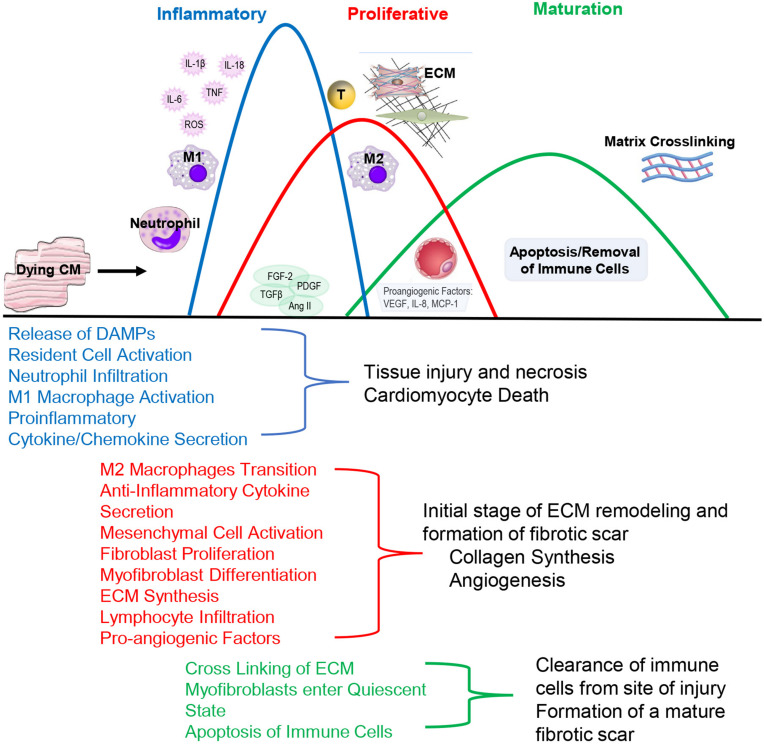
During the Inflammatory Phase, damage or death of cardiomyocytes activates resident fibroblasts and other cell populations to produce cytokines and recruit pro-inflammatory leukocyte populations, primarily neutrophils and monocytes that differentiate into M1-like macrophages. This acute, pro-inflammatory response transitions into a reparative response during the Proliferative Phase with the recruitment of reparative lymphocyte and M2-like macrophages populations. Activated myofibroblasts secrete collagen and other extracellular matrix components. Pro-angiogenic factors are secreted that promote neovascularization. The events conclude during the Maturation Phase with the apoptosis of reparative immune cell and myofibroblast populations and matrix crosslinking, resulting in mature scar formation.

Pathologically, inflammation regulates virtually all aspects of cardiovascular health including cardiomyocyte contractility and cardiac fibrosis and represents an important regulatory mechanism. Following injury, acute inflammatory responses occur that help remove dead or damaged cardiomyocytes, ECM debris and initiate healing. Cardiac repair after injury is a finely tuned and regulated series of events that is critical for adequate healing (Figure 2). With the exception of myocarditis, other forms of cardiac injury are considered sterile inflammation and follow a similar series of events. This progression of events has been well defined for myocardial infarction and include the inflammatory, proliferative and maturation phases (Prabhu and Frangogiannis, 2016). While these responses may not be identical in timing, duration and magnitude between heart failure etiologies, they are thought to be broadly applicable to other forms of sterile cardiac damage (Fildes et al., 2009). Following insult or injury, there is an acute inflammatory phase characterized by infiltration of pro-inflammatory immune cell populations that digest and clear damaged cells and ECM tissue. This is followed by a reparative phase with the resolution of pro-inflammatory responses and activation of reparative responses such as myofibroblast accumulation, ECM deposition and neovascularization. Appropriate magnitude and duration of each event is critical for optimal repair. Early inflammatory activation is needed for the transition to a reparative response whereas an excessive inflammatory phase can further tissue damage and lead to improper healing.

## The Inflammatory Phase

**FIGURE 3:**
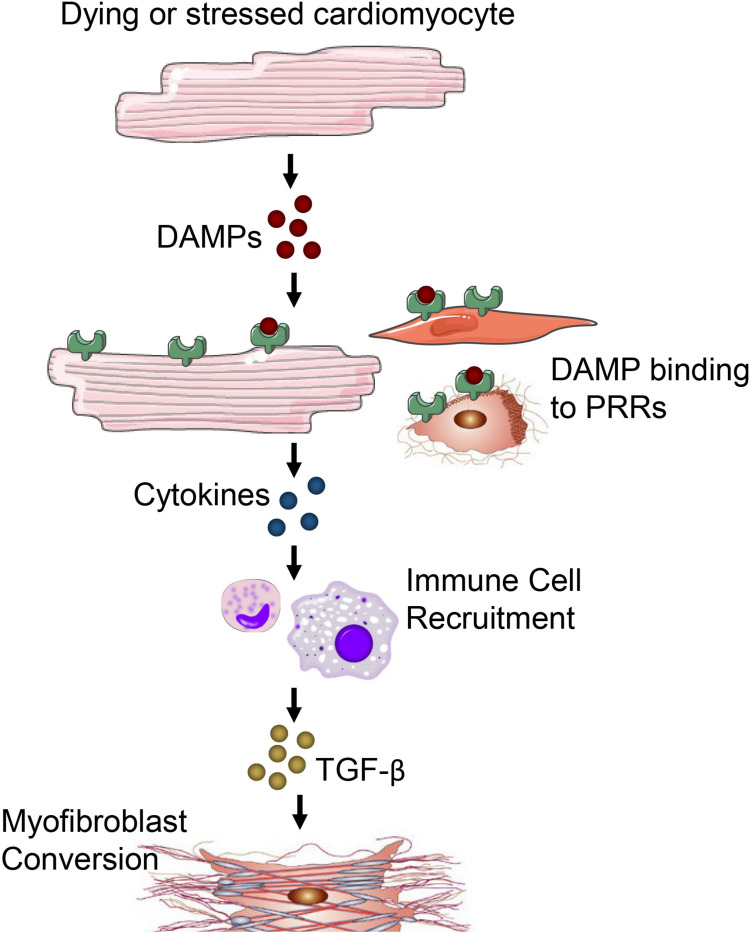
Damage or death of cardiomyocytes leads to the release of DAMPs. DAMPs acting through PRRs on neighboring cardiomyocytes, fibroblasts, resident immune cells, endothelial cells and other parenchymal cells of the heart promote cytokine and chemokine release. Immune cells migrate in response to cytokine/chemokines in the heart and secrete TGF-β and other pro-fibrotic factors to promote fibrosis and myofibroblast conversion.

The inflammatory phase is characterized by the recruitment of inflammatory cells to the site of damage (Prabhu and Frangogiannis, 2016). Cardiomyocytes are more susceptible to ischemic injury or damage than non-cardiomyocytes. Injury or death of cardiomyocytes causes the release of danger-associated molecular patterns (DAMPs) that bind to a cognate pattern recognition receptor (PRR) on neighboring cells to initiate inflammatory responses (Figure 3). A number of factors released from damaged or dying cardiomyocytes have been identified as DAMPs including mitochondrial DNA (Bliksoen et al., 2016), the chromatin protein high mobility group box 1 (HMGB1) (Lotze and Tracey, 2005), purine metabolites (Kono et al., 2010; McDonald et al., 2010), sarcomeric protein fragments (Lipps et al., 2016), and S100 proteins (Rohde et al., 2014). Additionally, fragments of the ECM that arise from damage including biglycan (Schaefer et al., 2005), decorin (Merline et al., 2011), hyaluronan (Scheibner et al., 2006) and fibronectin (Gondokaryono et al., 2007; Lefebvre et al., 2011; Sofat et al., 2012) have been shown to activate PRRs to contribute to inflammatory responses.

DAMP activation of PRRs induces the production of a cascade of inflammatory mediators including cytokines, chemokines and cell adhesion molecules (Mann, 2011). PRRs are present on cells of the innate immune system but can also act on surviving resident cell populations including cardiomyocytes (Kukielka et al., 1993; Frantz et al., 1999; Tarzami et al., 2002), fibroblasts (Zhang et al., 2015; Turner, 2016) and endothelial cells (Kumar et al., 1997) to potentiate the inflammatory cascade (Frantz et al., 1999). The best characterized family of PRRs are the Toll-like receptors (TLR) but others include nucleotide-binding oligomerization domain-like receptors (NLRs) and receptor for advanced glycation end-products (RAGE). Signal transduction by PRRs has been extensively delineated and converges on the activation of mitogen-activated protein kinases (MAPKs) and nuclear factor (NF)-κB to regulate the expression of a large panel of pro-inflammatory genes including cytokines, chemokines and adhesion receptors (Akira and Takeda, 2004). These factors enhance leukocyte recruitment to amplify the inflammatory response, promote efferocytosis of dying cells and augment tissue digestion by proteases and oxidases.

Due to their close proximity, wide distribution in the myocardium, ability to rapidly respond to stimuli and potential as a source of inflammatory mediators, fibroblasts have been proposed to serve as sentinel cells in the heart. Within days of tissue damage, cardiac fibroblasts acquire a pro-inflammatory phenotype characterized by the secretion of cytokines/chemokines including IL-8, IL-1β, CCL2, eotaxin and TNF-α and the presence of matrix-degrading properties (Sandanger et al., 2013; Sandstedt et al., 2019). The inflammatory potential of cardiac fibroblasts has been extensively documented *in vitro* (Heim et al., 2000; Lafontant et al., 2006). Stimulation of cardiac fibroblasts with ATP results in a large release of pro-inflammatory cytokines (Lu et al., 2012). A number of different factors in addition to ATP are also known to cause fibroblast activation including reactive oxygen species (ROS) (Siwik et al., 2001; Lijnen et al., 2006; Lu et al., 2012) and cytokines (Lafontant et al., 2006; Zymek et al., 2007; Turner et al., 2009). Cytokines have been implicated in inducing an inflammatory phenotype in cardiac fibroblasts and potentiating cytokine and chemokine synthesis (Lafontant et al., 2006; Zymek et al., 2007; Turner et al., 2009). They have also been shown to regulate expression of matrix-degrading proteases (Li et al., 2002; Siwik and Colucci, 2004). However, the contribution of cardiac fibroblasts in activating inflammatory cascades in pathological settings is less understood. *In vivo* studies have been limited due to the absence of specific markers for cardiac fibroblasts (Kong et al., 2013). As a result, studies have been largely descriptive. However, infarction models in mice show activation of the inflammasome in cardiac fibroblasts, an indication of the generation of active IL-1β (Kawaguchi et al., 2011; Sandanger et al., 2013).

Endothelial and resident mast cell populations have also been implicated in triggering the inflammatory cascade post-infarction (Lakshminarayanan et al., 1997, 2001; Frangogiannis et al., 1998a). As previously mentioned, there is a small population of resident mast cells that plays an important role in homeostasis in the normal myocardium and during pathological events. Expansion of the mast cell population is associated with cardiac fibrosis in response to multiple pathological challenges (Frangogiannis et al., 1998b; Patella et al., 1998; Shiota et al., 2003; Wei et al., 2003). The mechanisms associated with this expansion is not well understood. Stem cell factor (SCF), which is known to be involved in the recruitment and differentiation of mast cell progenitors, is upregulated in hearts following myocardial infarction and may contribute to the proliferation of resident mast cells (Frangogiannis et al., 1998b). However, other studies suggest mast cell progenitors infiltrate the myocardium from outside sources (Bujak et al., 2008). Regardless of origin, mast cells are known to be vital in the pathogenesis of cardiac fibrosis. Mast cell deficiency results in attenuated perivascular fibrosis and reduced progression to decompensated heart failure in a mouse model of pressure overload (Hara et al., 2002). Pharmacological prevention mast cell product release in spontaneously hypertensive rats reduced fibrosis, reduced inflammatory cell recruitment and decreased pro-inflammatory cytokines (Levick et al., 2009).

How mast cells influence fibrosis is also poorly understood. Mast cells are known to have abundant numbers of granules that store a wide range of mediators. This includes many pro-fibrotic mediators including TNF-α (Frangogiannis et al., 1998a), TGF-β (Shiota et al., 2003), and platelet-derived growth factor (PDGF) (Nazari et al., 2016). However, these mediators are produced by many cell types and the relative contribution of mast cells has not been fully elucidated. Additionally, mast cells have abundant expression of chymase, a protease implemented in the angiotensin converting enzyme (ACE)-independent generation of angiotensin II (Urata et al., 1990a, b). This mechanism may represent an important mechanism in the progression of cardiac fibrosis in the presence of ACE inhibition.

The cytokine rich environment present in the heart following injury causes infiltration of pro-inflammatory immune cell populations including phagocytic neutrophils and mononuclear cells which clear the area of dead cells and ECM debris (Prabhu and Frangogiannis, 2016). These responses are facilitated by changes in the vasculature. Hypoxia compromises the vascular endothelial cell integrity and barrier function, increasing vessel permeability to facilitate leukocyte infiltration (Sansbury and Spite, 2016). Neutrophils are among the first immune cell types to infiltrate into the damaged heart in response to a number of pro-inflammatory mediators including DAMPs, cytokines, chemokines, endogenous lipid mediators (prostaglandins and leukotrienes), histamine and complement components (Yan et al., 2013; Puhl and Steffens, 2019). Neutrophils are continually produced from hematopoietic progenitors in the bone marrow through the process of granulopoiesis. They reside in specific niches in the bone marrow through the action of CXCL12 (Katayama et al., 2006; Russell et al., 2011). Maturation of immature neutrophils is regulated by granulocyte colony-stimulating factor (G-CSF), which is produced in response to IL-17 from γδT-cells and counteracted by IL-23 (Stark et al., 2005; Liao et al., 2012; Yan et al., 2012; Savvatis et al., 2014). In this way γδT-cells regulate neutrophil and macrophage infiltration and have detrimental effects on remodeling in myocardial infarction models (Yan et al., 2012). Following maturation, neutrophils remain in the bone marrow through the actions of CXCR4 or are release into the circulation by CXCR2-dependent signaling (Tarzami et al., 2003; Devi et al., 2013).

Extravasation of neutrophils into the heart is dependent on adhesion interactions between the neutrophils and endothelial cells. Endothelial cells activated by PRR-dependent mechanism rapidly upregulate pre-stored *P*-selectin. There is a slower upregulation of *E*-selectin that is generated *de novo* (Ley et al., 2007; Petri et al., 2008). Circulating neutrophils express selectin ligands, which causes them to interact with the endothelium and roll along the endothelial layer. The two selectins have partially overlapping functions and bind P-selectin glycoprotein ligand 1 (PSGL1) leading to tethering of neutrophils and initiate rolling (Ley et al., 2007). Lymphocyte function-associated antigen 1 (LFA1), which binds to intracellular adhesion molecule (ICAM) 1 and 2 on endothelium facilitates neutrophil rolling (Zarbock et al., 2011). Rolling neutrophils respond to chemokines bound to the endothelial surface to induce a conformational change of integrins and endothelial cell surface molecules such as ICAM1 and ICAM2, enhancing their adhesion and resulting in arrest (Detmers et al., 1990; Herter and Zarbock, 2013). It is thought that full activation requires a two-step process initiated by specific priming pro-inflammatory cytokines including TNFα and IL-1β however other chemoattractants and growth factors may also be involved (Summers et al., 2010). This priming is also important for maximal neutrophil degranulation and activation (Guthrie et al., 1984; Summers et al., 2010). Signaling initiated by CXCL8 in humans (CXCL1, CXCL2 and CXCL5 in mice) via CXCR2 further activates neutrophils and promotes their adhesion (Pruenster et al., 2009; Williams et al., 2011). The neutrophils transmigrate through the endothelial junctions and then the basement membrane through multiple effectors including VCAM1, PECAM1, and VLA4 (Wang et al., 2006). Many of these processes have been studied extensively in other tissues and are believed to be directly applicable to the heart, however, a careful examination of cardiac specific neutrophil extravasation and transmigration process have not been extensively investigated.

Once in the tissue, neutrophils release proteolytic enzymes such as myeloperoxidase (MPO) and play an important role in clearing the area of dead cells and matrix debris (Puhl and Steffens, 2019). They may also amplify the immune response through production of pro-inflammatory mediators (Boufenzer et al., 2015) and have been shown to regulate recruitment of pro-inflammatory monocyte populations to the heart (Alard et al., 2015). While these actions are critical for proper healing, neutrophils may also exert cytotoxic actions on cardiomyocytes to exacerbate the injury (Simpson et al., 1988; Entman et al., 1992; Ali et al., 2016). These cytotoxic effects occur through the release of reactive oxygen species (ROS) and also release of granules associated with adverse left ventricular remodeling (Vasilyev et al., 2005; Ciz et al., 2012).

Following injury, this resident macrophage populations expands (Yan et al., 2013). There are two distinct subsets of monocyte recruitment to the damaged heart (Nahrendorf et al., 2007). Pro-inflammatory, M1-like macrophages are recruited to the heart shortly after neutrophils. This initial population of macrophages is derived from bone marrow progenitor cells and release from splenic reservoirs. They are recruited to the heart through the MCP-1(CCL2)/CCR2 axis (Swirski et al., 2009; Bajpai et al., 2018). The first subset is pro-inflammatory and recruited through the MCP-1(CCL2)/CCR2 axis. This pro-inflammatory population is characterized by high Ly-6C expression (Dewald et al., 2005). Infiltrating Ly-6C^*high*^ populations are derived from bone marrow progenitor cells and reservoirs of mononuclear cells in the spleen that can be deployed quickly to the site of inflammation (Swirski et al., 2009). These M1-like macrophages are proteolytic with increased expression of proteinases such as cathepsins and MMPs and are involved in ECM remodeling due to being a major source of MMPs and TIMPs (Huang et al., 2012; Khokha et al., 2013). Like fibroblasts, macrophages play a role in ECM remodeling through the secretion of ECM components. These classically activated macrophage populations serve as a major source of pro-inflammatory cytokines including IL-12, IL-23, IL-1, and IL-6 as well as being involved in phagocytosis.

## The Proliferative Phase

Suppression and resolution of inflammation is an active process. While the mechanisms contributing to the initiation of inflammation have been well characterized, resolution of inflammation is not as well understood. Neutrophils that are recruited initially during the inflammatory phase are short-lived cells and rapidly undergo cell death primarily through apoptosis, but also necrosis, which releases mediators that promote the resolution of inflammation including lipoxins and resolvins that suppress neutrophil transmigration and promote neutrophil apoptosis (Serhan et al., 2008; Mantovani et al., 2011; Geering et al., 2013). Dying neutrophils also express decoy and scavenging receptors that deplete the area of inflammatory mediators (Soehnlein and Lindbom, 2010; Penberthy and Ravichandran, 2016). Phagocytosis of necrotic neutrophils by macrophages clears the area of apoptotic cells and induces a pro-resolving M2 macrophage phenotype characterized by the secretion of suppressors of inflammation such as transforming growth factor (TGF)-β, IL-10, interleukin receptor associated kinase-M (Chen et al., 2012) and pro-resolving lipid mediators such as lipoxins and resolvins (Sansbury and Spite, 2016; Horckmans et al., 2017). An anti-inflammatory/reparative monocyte subpopulation is recruited to contribute to the M2-like macrophage pool and contributes to the resolution of inflammatory responses. Similarly, these pro-resolving, M2-like macrophages secrete anti-inflammatory, pro-fibrotic and pro-angiogenic cytokines including IL-10 and TGF-β to suppress inflammation and promote tissue repair.

Dendritic cells infiltrate the damaged heart predominantly during the reparative phase (Yan et al., 2013). They play an important role in the resolution of inflammation, scar formation and angiogenesis. Deletion of dendritic cells prolongs the accumulation of Ly-6C^*high*^ monocytes, pro-inflammatory macrophages and pro-inflammatory mediators (Anzai et al., 2012). Mice lacking dendritic cells have a reduction in endothelial cell proliferation and worsened cardiac function following myocardial infarction. Additionally, they have been shown to play a role in activation of T cell populations, which play a role in remodeling (Van der Borght et al., 2017). They also play an important role in phagocytosis of foreign or damaged material and antigen presentation making them an important link between the innate and adaptive immune response (Dieterlen et al., 2016; Van der Borght et al., 2017).

Lymphocytes migrate to the heart following injury and there is emerging evidence for an important role of lymphocyte populations in mediating cardiac fibrosis in both ischemic and non-ischemic heart failure (Laroumanie et al., 2014; Nevers et al., 2015; Bansal et al., 2017). The cause of T lymphocytes in the non-ischemic myocardium is uncertain but may be a result of mechanical-stress activation of neurohumoral pathways (Amador et al., 2014; Li et al., 2017). In the ischemic heart, T cells are recruited via chemokine-dependent mechanisms primarily during the reparative phase (Dobaczewski et al., 2010b). Cytotoxic T cells are activated after infarction and may exert cytotoxic actions on healthy cardiomyocytes in a mechanism that is thought to involve cross-reactive cardiac antigens (Varda-Bloom et al., 2000; Ilatovskaya et al., 2019). B cells are also recruited to the heart through poorly understood mechanisms (Wang et al., 2019). They are thought to have a negative impact on remodeling though their role is not well defined (Adamo et al., 2018, 2020). B cells promote mobilization of pro-inflammatory Ly-6C^*high*^ monocytes through the production of CCL7 and may affect the heart through their role in antibody deposition (Zouggari et al., 2013; Adamo et al., 2020). CD4+ helper T cells play an important role in response to cardiac injury. Following myocardial infarction, they are likely activated by cardiac autoantigens to promote wound healing, resolution of inflammation, proper collagen matrix and scar formation (Hofmann et al., 2012). Studies using CD4+ T-cell deficient mice, mice lacking the MHCII genes and OT-II mice that have defective T-cell antigen recognition, have augmented infiltrating leukocytes and disrupted collagen matrix formation Hofmann et al., 2012). Regulatory T cells (CD4+ Foxp3+) are also critical for favorable wound healing, scar formation and resolution of inflammation after myocardial infarction, in part, through modulating macrophage polarization toward an M2-like phenotype (Weirather et al., 2014). NKT cell activation reduces leukocyte infiltration and adverse remodeling following both non-perfused and reperfused myocardial infarction partially through enhanced expression of IL-10 and other anti-inflammatory cytokines (Sobirin et al., 2012; Homma et al., 2013).

Along with the repression of inflammation, there is induction of mediators that activate mesenchymal cells. During the proliferative phase there is abundant infiltration of fibroblasts and vascular cells. Suppression of pro-inflammatory signaling such as IL-1β and interferon-γ-inducible protein (IP)-10 allows for growth and infiltration of cardiac fibroblasts (Palmer et al., 1995; Koudssi et al., 1998). Fibroblast migration is a critical aspect of fibroblast biology in the damaged myocardium. Fibroblasts must migrate to the site of dead cardiomyocytes for optimal repair in a manner that is dependent on their ability to degrade and deposit matrix (Tschumperlin, 2013). Several factors have been shown to mediate fibroblast migration including leukotrienes (Blomer et al., 2013), cytokines such as IL-1β and cardiotrophin-1 (Mitchell et al., 2007; Freed et al., 2011) and growth factors including fibroblast growth factor (FGF) and TGF-β (Detillieux et al., 2003; Liu et al., 2008).

Fibroblast proliferation also plays an important role during the proliferative phase. Studies demonstrate an intense proliferation of fibroblasts in the injured heart (Frangogiannis et al., 1998b; Virag and Murry, 2003). Many factors influence fibroblast proliferation including the growth factors fibroblast growth factor (FGF)-2 and platelet-derived growth factor (PDGF) (Booz and Baker, 1995; Zymek et al., 2006). Other factors including angiotensin II, mast cell-derived tryptase and chymase also play a role. However, the relative importance of these factors is not well defined.

Following infiltration and proliferation at the sight of injury, fibroblasts differentiate into myofibroblast. Myofibroblasts arise primarily through proliferation of resident fibroblasts (Fredj et al., 2005; Teekakirikul et al., 2010; Ali et al., 2014; Moore-Morris et al., 2014) and are characterized by the expression of contractile proteins and the ability to secrete large amounts of matrix proteins (Cleutjens et al., 1995b). While myofibroblasts may become activated through several potential mechanisms, transforming growth factor (TGF)-β is the best characterized mechanism of myofibroblast activation. TGF-β is upregulated in the damaged heart and induces transcription of myofibroblast genes through canonical Smad-dependent signaling (Dobaczewski et al., 2010a). Alternatively, non-canonical signaling through p38 mitogen-activated protein kinase (MAPK), also plays a role in myofibroblast conversion (Hashimoto et al., 2001; Sousa et al., 2007).

Activated myofibroblasts secrete ECM to form the fibrotic scar. Myofibroblasts are thought to represent the main source of ECM deposition (Cleutjens et al., 1995a; Squires et al., 2005). Secretion of structural proteins including collagens and fibronectin as well as matrix metabolism through the expression of MMPs and TIMPs are critical for fibrosis. At the end of the proliferative phase, there is an ECM composed primarily of collagen. Signals leading to the transition from the proliferative to the maturation phase are not well characterized. Regardless, fibrotic and angiogenic responses are halted, preventing the expansion of fibrosis and leading to the maturation phase.

## The Maturation Phase

The maturation phase follows the proliferative phase and is characterized by mature scar formation. During the maturation phase, cross-linking of the extra cellular matrix occurs. Reparative cells that are present during the proliferative phase become deactivated and may go through apoptosis. The mechanisms involved in the transition from the proliferative phase to the maturation phase are largely unknown. Myofibroblasts undergo quiescence, potentially due to a lessening of fibrotic growth factors and decreased TGF-β and angiotensin II signaling. They may also go through apoptotic death (Takemura et al., 1998).

## Inflammation in Myocarditis

Inflammatory cardiomyopathy or myocarditis occurs due to inflammation in the heart. Unlike other forms of heart failure, myocarditis is initiated by a pathogen or autoimmune response and may produce a unique type of inflammation depending on the causative agent. Myocarditis is commonly associated with viral infection (Caforio et al., 2007), the Coxsackie virus of group B (CVB) is the most studied, which leads to viral particle processing by innate immune cells followed by antigen presentation and activation of the antiviral cytotoxic CD8+ T cells and some CD4+ T cell populations. While viruses of the adenovirus, enterovirus, and parvovirus families are most commonly associated with myocarditis, other infectious events including bacterial causes such as staphylococcus, streptococcus and Clostridia infections, fungal diseases including aspergillosis and actinomycosis, protozoan illnesses such as Chagas disease and malaria and parasitic infections like in schistosomiasis. Toxins and autoimmune disorders can also give rise to myocarditis (Caforio et al., 2007). While the immune response is unique depending on the cause of myocarditis, common hallmarks include inflammatory cell infiltration, which can lead to fibrosis. Inflammation in myocarditis is highly linked to the severity of the disease (Kindermann et al., 2008). Most individuals who have inflammatory cardiomyopathy see a resolution of symptoms however, the type, extent and duration of the inflammatory response determines whether myocarditis will be resolved or progress to dilated cardiomyopathy and ultimately heart failure. Patients with an acute hypersensitive myocarditis seem to recover after a few days, while patients with giant cell myocarditis and eosinophilia myocarditis more often progress to heart failure (Fung et al., 2016).

Similar to other forms of injury, the noxious insult initiating myocarditis causes damage to cardiomyocytes, stimulating the recruitment of circulating immune cells. If the extent of damage results in a loss of cardiomyocytes, the heart repairs itself through the deposition of ECM and myocardial fibrosis. This process can be exacerbated by continued inflammation due to prolonged exposure to the pathogen or toxic agent, T lymphocyte responses to specific antigens and persistent immune responses due to antibodies against or similar to endogenous heart antigens (Cooper, 2009).

## Inflammation in Hypertension

Hypertension involves both the innate and adaptive immune system throughout the progression of the disease. Inflammation is believed to be a contributing factor to the diseased state of hypertension. T lymphocytes have been shown to have a role in the onset of the disease in an Angiotensin II (Ang II)-induced hypertension mouse model. In a RAG-1^–/–^ mouse model null of T and B lymphocytes, mice display a dampened form of hypertension, while reintroduction of T cells recapitulated the classical hypertension readouts (Guzik et al., 2007). A more recent study closely investigated the role of B lymphocytes in Ang II-induced hypertension and found depletion of B cells ablated the phenotype associated with the model and adoptive transfer of B cells recapitulated the hypertensive phenotype (Chan et al., 2015). In a similar Ang II infusion induced hypertensive mouse model, immune cells of the monocytic lineage were shown to be a key mediator in enabling vascular dysfunction (Wenzel et al., 2011; Harrison, 2014). Ablation of LysM+ monocytes in this model significantly dampened vascular macrophage infiltration, aortic macrophage populations, and inflammatory gene expression in vasculature. The common accepted mechanism follows enhancement of hypertension symptoms due to the stress put on vasculature and the release of damage associated molecular patterns (DAMPs) causing a secondary chronic inflamed state (Drummond et al., 2019). The involvement of the immune system in hypertension further exacerbates the disease state induced by high blood pressure.

## Cytokines-Mediators of Fibrosis

Many of the Th-2 cytokines were first recognized as having pro-fibrotic properties including IL-4, IL-5, IL-10, and IL-13. IL-4 has been shown to increase collagen and matrix protein synthesis in fibroblasts (Fertin et al., 1991; Postlethwaite et al., 1992) and deletion reduces myocardial fibrosis (Kanellakis et al., 2012; Peng et al., 2015). IL-4 is pleiotropic in nature and effects a variety of cell types including having immunosuppressive effects on pro-inflammatory mediators (Hart et al., 1989; Levings and Schrader, 1999). IL-13 has also been shown to directly activate fibroblasts (Oriente et al., 2000) and plays a role in fibrosis and deletion in mice aggravates healing after myocardial infarction (Hofmann et al., 2014).

While many immune responses are thought to be a reciprocal regulation between cell populations, Th-1 and Th-17 cytokines also promote fibrogenesis (Mosmann and Coffman, 1989; Choy et al., 2015). IL-17 has reported direct and indirect pro-fibrotic properties (Li et al., 2014). Some Th1 cytokines, including TNF-α, are pro-fibrotic while others, such as IFN-γ and IL-12, are anti-fibrotic (Zhang et al., 2011; Han et al., 2012; Li et al., 2012; Kimura et al., 2018; Lee et al., 2019).

## Interleukin-1

IL-1α and IL-1β are upregulated in the injured heart and play an important role in inducing the expression of other cytokines, chemokines, adhesion molecules and growth factors (Guillen et al., 1995; Herskowitz et al., 1995). Most cell types in the heart are impacted by IL-1 family members, particularly IL-1β. It plays a large role in pro-inflammatory leukocyte recruitment to the heart following damage (Saxena et al., 2013). Notably, IL-1β has been found to be particularly important in regulating cardiac fibroblast function during the inflammatory phase. It is markedly upregulated in the infarcted myocardium (Herskowitz et al., 1995; Christia et al., 2013). Induction mediates inflammatory signaling and ECM metabolism through its effects on proteases (Bujak et al., 2008). IL-1β promotes fibroblast migration through increasing the expression of proteins involved in ECM turnover (Mitchell et al., 2007). IL-1β may also play a role in the conversion of fibroblasts into myofibroblasts (Saxena et al., 2013). It has also been linked to fibroblast proliferation where it has been shown to inhibit proliferation through the modulation of cyclins, cyclin-dependent kinases and their inhibitors (Palmer et al., 1995; Koudssi et al., 1998). However, many of these studies were performed *in vitro* and the actions of IL-1β *in vivo* is less clear.

*In vivo* studies assessing the role of IL-1β are more confounding. Viral overexpression of IL-1 receptor antagonist (IL-1ra) in the hearts of rats subjected to ischemia reperfusion injury was protective through the inhibition of inflammatory responses and decreased cardiomyocyte apoptosis (Suzuki et al., 2001). Global IL-1RI knockout mice have decreased inflammation and immune cell recruitment following myocardial infarction, which culminated in an attenuated fibrotic response (Bujak et al., 2008). Contrarily, neutralizing antibody administration for IL-1β in mice in the acute phase after myocardial infarction delayed the wound healing process leading to increased incidence of cardiac rupture and enhanced maladaptive remodeling long-term (Hwang et al., 2001). These confounding studies demonstrate the increasingly appreciated pleiotropic nature of many cytokines including the IL-1 family.

## Interleukin-6

IL-6 has been extensively characterized for its role in increasing fibroblast proliferation and myocardial fibrosis (Banerjee et al., 2009). It is a member of a family of structurally related cytokines including oncostatin-M and cartotrophin-1 that have overlapping functions. IL-6 effects most cells of the heart. In cardiomyocytes, IL-6 protects cells from death and promotes hypertrophy (Sano et al., 2000; Smart et al., 2006). Inhibition of IL-6 diminishes acute immune cell recruitment (Muller et al., 2014). Lack of IL-6 protects the heart from fibrosis in several models of heart failure (Gonzalez et al., 2015; Zhang et al., 2016).

## Interleukin-10

IL-10 is upregulated in the injured heart (Frangogiannis et al., 2000a). It is produced primarily by activated Th2 cells and monocytes that have anti-inflammatory properties (Frangogiannis et al., 2000a). In macrophages, IL-10 suppresses the synthesis of pro-inflammatory cytokines and chemokines such as IL-1, IL-6, and TNF-α (Fiorentino et al., 1991). It can also regulate ECM remodeling through the regulation of MMPs and TIMPs (Lacraz et al., 1995). IL-10 knockout mice have increased mortality and enhanced inflammation in response to ischemia/reperfusion injury (Yang et al., 2000). Since IL-10 is increased at later time points following myocardial infarction and has potent anti-inflammatory effects, it would be anticipated that IL-10 might play an important role in the resolution of inflammation. However, studies have been conflicting. Studies using IL-10 knockout mice showed that, while mice have augmented acute inflammatory responses, resolution of inflammation was unchanged (Zymek et al., 2007).

## Tumor-Necrosis Factor

TNF-α is a pleiotropic cytokine capable of effecting all cell types involved in cardiac injury and repair. It is able to suppress cardiac contractility and augment cardiomyocyte apoptosis (Finkel et al., 1992; Yokoyama et al., 1993). Secretion by various cell types involved in remodeling enhances production of pro-inflammatory cytokines, chemokines and adhesion molecules by immune cells. TNF-α can also effect ECM metabolism through its ability to decreased collagen synthesis in fibroblasts and enhance MMP activity (Siwik et al., 2000). While these findings and studies showing that TNF-α knockout mice have decreased inflammation and improvements in cardiac remodeling and function following myocardial infarction suggest TNF-α neutralization would be beneficial in the injured heart, this has not been the case (Maekawa et al., 2002; Berthonneche et al., 2004; Sugano et al., 2004; Sun et al., 2004). Inhibiting TNF-α through gene therapy to express the soluble TNF receptor produced deleterious effects in a mouse model of myocardial infarction through increased incidence of cardiac rupture and augmented cardiac remodeling (Monden et al., 2007b). Genetic deletion of TNFR1/TNFR2 produced increased infarct size and enhanced cardiomyocyte apoptosis following myocardial infarction (Kurrelmeyer et al., 2000). These findings have been proposed to be due to distinct TNF-α effects through different receptor subtypes (Monden et al., 2007a), but may also be attributed to the complex nature of TNF-α signaling on biological processes in the heart.

## Interferons

Interferons (IFN) can be secreted by immune cells or fibroblasts to effect a wide array of biological responses (Noppert et al., 2007; Ivashkiv and Donlin, 2014). IFN-γ knockout mice have a reduction in the myofibroblast marker α-smooth muscle actin following angiotensin II administration (Han et al., 2012). Similarly, mice lacking the INF-γ receptor have decreased cardiac hypertrophy and fibrosis along with reductions in macrophage and T cell infiltration following angiotensin II infusion (Marko et al., 2012). However, these studies used global knockout mice and fail to determine the specific cardiac contribution of immune cells compared to fibroblasts.

## Transforming Growth Factor Family

TGF-β1 has been proposed to be a master regulator in the transition from inflammation to repair in the damaged heart (Dobaczewski et al., 2011). Neutralization of TGF- β1 worsens cardiac dysfunction and prolongs inflammation in a model of myocardial infarction (Ikeuchi et al., 2004). However, cardiomyocyte-specific knockout of TGF-β receptors is protective and promotes anti-inflammatory and cytoprotective signaling (Rainer et al., 2014). These studies suggest that the detrimental effects of loss of TGF-β1 signaling is not likely through directly impacting cardiomyocytes, but though loss of anti-inflammatory actions and fibrosis. In addition to its role in suppressing inflammation and promoting reparative immune responses, TGF-β1 is critical for myofibroblast transdifferentiation (Hashimoto et al., 2001; Wang et al., 2005; Dobaczewski et al., 2010a).

Growth differentiation factor-15 (GDF-15) is also a member of the TGF-β family that has been implicated in suppression of inflammation after myocardial infarction. GDF-15 counteracts integrin activation on leukocytes to curb pro-inflammatory responses (Kempf et al., 2011). Knockout of GDF-15 in mice augments inflammation and increases cardiac rupture following myocardial infarction (Kempf et al., 2011). This is also reflected in the patient population where patients with elevated plasma GDF-15 are prone to increased mortality (Kempf et al., 2007).

## Clinical Perspectives

Currently approved heart failure therapies target the short-term clinical status to minimize symptoms and improve quality of life, but long-term prognosis remains poor (Machaj et al., 2019). Treatments aimed at preventing the progression of heart failure or reversing maladaptive remodeling are an attractive target, but have culminated in minimal success. Due to the involvement of inflammation in all stages of disease progression, targeting the immune response has been an ongoing area of interest to address this unmet clinical need. In the past two decades the field of cardiac inflammation has made numerous ventures into clinical trials targeting inflammatory pathways. To date, cytokine-targeted therapies have dominated with clinical trials targeting TNF-α and IL-1β (Murphy et al., 2020).

Early studies identified TNF-α as a potential therapeutic target due to its known role as a pro-inflammatory mediator in heart failure. To date, several randomized, placebo-controlled, anti-TNF-α studies have been performed. The RENEWAL trial (Randomized Etanercept Worldwide Evaluation), testing etanercept, and the ATTACH trial (Anti-TNF-α Therapy Against Congestive Heart Failure), involving infliximab, showed no indication of beneficial effects with treatment and the ATTACH trial exposed adverse effects of anti-TNF-α therapy (Bozkurt et al., 2001; Chung et al., 2003; Mann et al., 2004). These initial studies targeting the inflammatory response demonstrate our lack of understanding and under appreciation of the complexity of immune system involvement in heart failure. TNF-α is a widely expressed cytokine with pleotropic actions. This includes a protective role in cardiomyocytes by preventing death (Kurrelmeyer et al., 2000; Evans et al., 2018). Appropriate TNF-α levels may also be necessary for adequate tissue remodeling and repair (Anker and Coats, 2002).

IL-1β is another potential cytokine target for the treatment of heart failure due to its up-regulation in heart failure, role in pro-inflammatory responses and positive benefits from IL-1β inhibition in preclinical trials (Van Tassell et al., 2015). The CANTOS trial (Canakinumab Anti-Inflammatory Thrombosis Outcome Study) was a randomized, double-blinded, placebo-controlled, anti-IL-1β study investigating the use of the monoclonal antibody canakinumab (Ridker et al., 2017). All doses tested had a positive impact on inflammatory burden as indicated by C-reactive protein. However, only the highest dose examined (150 mg) was successful in reducing one of the primary end points of the study, reoccurrence of non-fatal MI, with no changes in other end points including stroke or cardiovascular death.

The ACCLAIM trial (Advanced Chronic Heart Failure Clinical Assessment of Immune Modulation Therapy) was a double-blinded, placebo-controlled study using a device-based non-specific immunomodulation therapy approach (Torre-Amione et al., 2008). This study did not find a significant reduction in cardiovascular hospitalization or mortality. However, in certain populations, those without a previous myocardial infarction and New York Heart Association (NYHA) II heart failure, significant reductions in primary endpoints were observed, suggesting that this approach may be beneficial in certain groups. Important to note, the mechanisms of immunomodulation are not well defined and the impact on cytokine levels were not measured making the findings difficult to interpret at a mechanistic level.

The clinical trials targeting inflammation for the treatment of heart failure, with the exception of the CANTOS trial (anti-IL-1β), have been largely disappointing (Van Tassell et al., 2015). However, to date, strategies have broadly targeted inflammation through either a generic approach or inhibition of cytokines that have an array of functions on many cell types. It is also important to note that these studies involve subsets of heart failure patients and it should not be discounted that these approaches may be valuable in certain patient populations such as inflammatory cardiomyopathies. These findings highlight our need for a better understanding of how inflammation contributes to the pathogenesis of heart failure. More recent preclinicial studies targeting a specific signaling pathways and cell populations give hope for future immunomodulatory therapies for the treatment of heart failure. The CCL2/CCR2 axis that is important for infiltrating pro-inflammatory monocytes has be targeting using small molecular antagonists (Hilgendorf et al., 2014; Grisanti et al., 2016; Liao et al., 2018; Patel et al., 2018), antibodies (Patel et al., 2018), small interfering RNA (Leuschner et al., 2011), lipid micelles containing CCR2 antagonists (Wang et al., 2018), and microparticles (Getts et al., 2014). Antibody-depletion based approaches targeting lymphocyte populations including CD3 and CD4 antibodies for T cell (Nevers et al., 2015; Bansal et al., 2017), CD25 for regulatory T cell (Bansal et al., 2019) and CD22 for B cell (Cordero-Reyes et al., 2016) depletion all display promise. However, if these and other preclinical studies translate in humans remains to be seen.

## The Impact of Inflammation on Fibrosis

**FIGURE 4:**
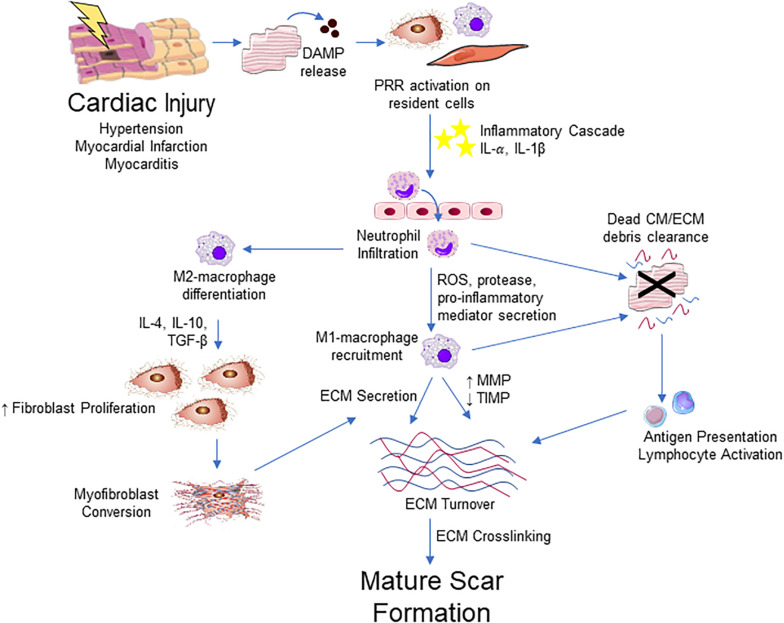
Integrative schematic of inflammation and cardiac fibrosis. After a cardiac insult, the release of DAMPs from dying or damaged cardiomyocytes (CM) triggers an inflammatory cascade through PRRs expressed on resident fibroblast, endothelial, mast cell, macrophage and other immune cell populations in the heart. This results in the cytokine/chemokine-mediated infiltration of the immune cells, initially neutrophils then Ly-6C^*high*^ monocytes that contribute to an increase in M1-like macrophages into the myocardium. M1-like macrophages and neutrophils contribute to clearance of dead cells and ECM debris through phagocytosis. Macrophages also contribute to ECM remodeling through the production of collagen and other ECM components and ECM turnover though the regulation of MMPs and TIMPs. Macrophage engulfment of apoptotic neutrophils promotes and M2-like phenotype, which promotes the proliferation and migration of fibroblast and promotes differentiation into myofibroblast, primarily through the action of TGF-β. Antigen presentation by phagocytic cells activate T lymphocyte populations that contribute to fibrosis through poorly defined mechanisms. Myofibroblasts are the major source of ECM components and contribute to remodeling of the ECM. The fibrotic response concludes with ECM crosslinking and the apoptosis or quiescence of immune cells and myofibroblasts, producing a mature scar.

It should be apparent that inflammation is a major regulator of the reparative response after cardiac injury (Figure 4). Inflammatory cells, such as neutrophils and macrophages, infiltrate to the site of injury where they release numerous pro-inflammatory mediators including tumor necrosis factor (TNF)-α, interleukin (IL)-1β and IL-6. These cytokines play an important role in the induction of resident fibroblast proliferation and activation of myofibroblasts initiating the production of ECM components (Christia et al., 2013). In turn, activated cardiac fibroblasts upregulate various cytokine and growth factors to influence healing via autocrine and paracrine-dependent mechanisms (Zhao and Eghbali-Webb, 2001).

Monocytes/macrophages play an important role in the fibrotic response following injury. While it is now recognized that the historical view of macrophage polarization into M1 and M2 phenotypes is an oversimplification and there are several differentiation states that are dynamic in response to changes in the environmental milieu (Xue et al., 2014), these distinct populations play unique roles in their modulation of fibrosis. Macrophage-derived TGF-β induces migration, growth and activation of fibroblasts and promotes collagen synthesis (Fine and Goldstein, 1987; Clark et al., 1997; Acharya et al., 2008). Macrophages also represent an important source of MMPs and TIMPs that influence matrix degradation (Huang et al., 2012; Khokha et al., 2013). MMPs are also involved in the control of chemokines to influence inflammatory responses (Van den Steen et al., 2000; Dean et al., 2008; Song et al., 2013).

During the proliferative phase of cardiac repair, cardiac fibroblast populations undergo expansion and conversion to myofibroblasts (Travers et al., 2016). The conversion of macrophages to a reparative phenotype promotes the recruitment and expansion of fibroblasts through the secretion of pro-fibrotic factors (Bajpai et al., 2018). These macrophages and recruited lymphocyte populations contribute to the activation of fibroblasts into a myofibroblast phenotype through cytokine release of factors such as TGF-β (Dobaczewski et al., 2011). Myofibroblasts, in turn, generate large amounts of ECM to repair the damaged heart (Travers et al., 2016).

The interplay between the immune and fibrotic response is extremely interconnected. Immune cells regulate all aspects of fibroblast biology and fibroblasts, in turn, regulate immune cell recruitment, activation and function. Dissecting the role of the various mediators has proven to be difficult due to the pleiotropic nature of many of these factors and their context-dependent and cell type-dependent nature. Furthermore, lack of cell type specific markers has hampered progress. Understanding the relationship between inflammation and cardiac remodeling is an important avenue of study due to its importance for recovery and represent a significant potential area of therapeutic intervention.

## Author Contributions

TT and LG designed the topic, collected the references, wrote the text, and revised the manuscript. Both authors contributed to the article and approved the submitted version.

## Conflict of Interest

The authors declare that the research was conducted in the absence of any commercial or financial relationships that could be construed as a potential conflict of interest.
